# LD block disorder-specific pleiotropic roles of novel *CRHR1* in type 2 diabetes and depression disorder comorbidity

**DOI:** 10.1007/s00406-023-01710-x

**Published:** 2023-12-14

**Authors:** Laura del Bosque-Plata, Mutaz Amin, Ricardo González-Ramírez, Rongling Wu, Teodor T. Postolache, Michael Vergare, Derek Gordon, Claudia Gragnoli

**Affiliations:** 1 Nutrigenetics, and Nutrigenomic Laboratory, National Institute of Genomic Medicine, 14610 Mexico City, Mexico; 2 INSERM US14-Orphanet, University of Paris, 75014 Paris, France; 3 Department of Biochemistry and Molecular Biology, Faculty of Medicine, Al-Neelain University, 11121 Khartoum, Sudan; 4 Department of Molecular Biology and Histocompatibility, “Dr. Manuel Gea González” General Hospital, 14080 Mexico City, Mexico; 5 Department of Public Health Sciences and Department of Statistics, Penn State College of Medicine, Hershey, PA 17033 USA; 6 Mood and Anxiety Program, Department of Psychiatry, University of Maryland School of Medicine, Baltimore, MD 21201 USA; 7 Rocky Mountain Mental Illness Research Education and Clinical Center (MIRECC), Veterans Integrated Service Network (VISN) 19, Military and Veteran Microbiome: Consortium for Research and Education (MVM-CoRE), Denver, CO 80246 USA; 8 Mental Illness Research Education and Clinical Center (MIRECC), Veterans Integrated Service Network (VISN) 5, VA Capitol Health Care Network, Baltimore, MD 21090 USA; 9 Department of Psychiatry and Human Behavior, Sidney Kimmel Medical College, Thomas JeffersonUniversity, Philadelphia, PA 19107 USA; 10 Department of Genetics, Rutgers University, Piscataway, NJ 08854 USA; 11 Department of Public Health Sciences, Penn State College of Medicine, Hershey, PA 17033 USA; 12 Division of Endocrinology, Department of Medicine, Sidney Kimmel Medical College, Thomas Jefferson University, Philadelphia, PA 19107 USA; 13 Division of Endocrinology, Department of Medicine, Creighton University School of Medicine, Omaha, NE 68124 USA; 14 Molecular Biology Laboratory, Bios Biotech Multi-Diagnostic Health Center, 00197 Rome, Italy

**Keywords:** *CRHR1 *gene Corticotropin-releasing hormone receptor 1 gene, T2D Type 2 diabetes, MDD Major depressive disorder, Linkage, LD Block, Linkage disequilibrium (LD), GR-α Glucocorticoid receptor-alpha, HLTF Helicase-like transcription factor, SMARCA3, MBNL1 Muscleblind-like splicing regulator 1

## Abstract

Major depressive disorder (MDD) and type 2 diabetes (T2D) are complex disorders whose comorbidity can be due to hypercortisolism and may be explained by dysfunction of the corticotropin-releasing hormone receptor 1 (CRHR1) and cortisol feedback within the hypothalamic–pituitary–adrenal axis (HPA axis). To investigate the role of the *CRHR1* gene in familial T2D, MDD, and MDD-T2D comorbidity, we tested 152 CRHR1 single-nucleotide-polymorphisms (SNPs), via 2-point parametric linkage and linkage disequilibrium (LD; i.e., association) analyses using 4 models, in 212 peninsular families with T2D and MDD. We detected linkage/LD/association to/with MDD and T2D with 122 (116 novel) SNPs. MDD and T2D had 4 and 3 disorder-specific novel risk LD blocks, respectively, whose risk variants reciprocally confirm one another. Comorbidity was conferred by 3 novel independent SNPs. In silico analyses reported novel functional changes, including the binding site of glucocorticoid receptor-alpha [GR-α] on *CRHR1* for transcription regulation. This is the first report of *CRHR1* pleiotropic linkage/LD/association with peninsular familial MDD and T2D. *CRHR1* contribution to MDD is stronger than to T2D and may antecede T2D onset. Our findings suggest a new molecular-based clinical entity of MDD-T2D and should be replicated in other ethnic groups.

**Supplementary Information:**

The online version contains supplementary material available at 10.1007/s00406-023-01710-x.

## Introduction

Major depressive disorder (MDD) and type 2 diabetes (T2D) are two prevalent complex diseases that share many pathogenic mechanisms and are often comorbid [1, 2]. MDD confers a 60% increase in T2D risk [3], and this elevated risk cannot be attributed to antidepressant therapy alone since T2D can occur in MDD drug-naïve subjects [4]. Furthermore, depressive symptoms in T2D patients are associated with higher morbidity and mortality [5]. Conversely, T2D is associated with a modest increase in MDD, and the latter appears to be driving the increased MDD-T2D comorbidity risk [3].

The hypothalamic–pituitary–adrenal axis (HPA axis) is a neuro-endocrine system modulating the stress response via interactions with serotonergic, noradrenergic, and dopaminergic brain systems [6]. The serotonergic and HPA systems are functionally interconnected and implicated in MDD [7–14], and they may play a role in T2D and associated metabolic traits [7, 12, 15–18]. It is unlikely that a single pathway or a few genes in a specific pathway will explain any one of these disorders or their comorbidity in all patients.

Under stress, the HPA axis releases corticotrophin-releasing hormone (CRH, alias corticotropin-releasing factor, CRF) from the hypothalamic paraventricular nucleus (PVN) to stimulate adrenocorticotropin (ACTH) secretion via the CRH receptor (CRHR) from the anterior pituitary, thereby elevating blood cortisol level. CRHR1 and CRHR2 are G-protein-coupled receptors [19], binding CRH and thereby mediating the production of ACTH, which triggers adrenal cortisol secretion. CRHR1 and CRHR2 mediate the axis responsiveness to integrated signals from diurnal rhythms, cortisol negative feedback, and superimposed stressors [20]. Preferentially, CRH binds CRHR1 and urocortin binds CRHR2, with a 40-fold affinity compared to CRH [21]. *CRHR1* is primarily expressed in the brain, most abundantly in the hippocampus, and also in the liver and adipose tissue, whereas *CRHR2* is primarily expressed in peripheral tissues (e.g., liver and adipose tissue) [21].

The *CRHR1* gene has been studied with depression for more than a decade [22, 23]. Several *CRHR1* variants and haplotypes have been linked to MDD in abused children (22) and to suicidal risk in adult males [24]. To our knowledge, there are no studies in human *CRHR1*-related T2D. However, the *CRHR1* 17q12 locus is linked to T2D [25] and metabolic syndrome [26]. CRHR1 stimulates beta-cell proliferation and insulin secretion in a glucose-dependent manner [27]. Thus, CRHR1 dysfunction may lead to hyperglycemia and T2D.

We hypothesize that hyperactivation of the neuro-endocrine CRH-norepinephrine (NE)-CRH system (CRH-NE-CRH), mediated by *CRHR1* variants, underlies MDD and may increase the risk for T2D, and thus, inherited *CRHR1* variants involved in stress responses might account, at least in part, for the comorbidity of MDD with T2D [28, 29].

In this study, we aimed to test for the first time whether the *CRHR1* gene, whose protein mediates the stress response, is linked to and/or in linkage disequilibrium (LD), understanding that LD is association, with MDD, T2D, and MDD-T2D comorbidity in very homogeneous well-characterized Italian families with T2D and positive familial T2D history and the presence of MDD, thus carrying the potential for the genetic comorbidity of these clinically associated disorders.

## Materials and methods

This study was performed using a previously published dataset [30, 31]: 212 deidentified homogeneous families from central Italy; descended from at least 3 generations of Italians, and with previously excluded families with uncertain ancestry and identical twins; with T2D and enriched familial history of T2D; and characterized for the presence or absence of lifetime MDD (DSM-IV criteria). Families were previously recruited following the Helsinki Declaration guidelines, and participants provided written informed consent prior to participation. This study was approved by the Thomas Jefferson University Ethical Committee.

In these families, 152 *CRHR1* single-nucleotide polymorphisms (SNPs) were amplified by microarray. Genotyping error exclusion was performed using PLINK [32].

Using the software Pseudomarker [33], we analyzed the data for 2-point parametric linkage to and linkage disequilibrium (LD; i.e., association), with T2D and MDD, using the models of dominant complete penetrance (D1) and recessive complete penetrance (R1). In a secondary analysis, we ran the dominant incomplete penetrance (D2) and recessive incomplete penetrance (R2) models.

Using the LD matrix (https://ldlink.nci.nih.gov/?tab=ldmatrix), we tested the absence or presence of LD blocks (defined by SNPs correlation r’ ≥ 0.9) within the analyzed variants. We compared the LD blocks containing the significant variants for MDD with the LD blocks containing the significant variants for T2D. We annotated the independent variants (i.e., not present within any LD block), that were significant for MDD, T2D, or both.

### In Silico functional predictions

We analyzed the *CRHR1* detected variants using different bioinformatics tools that predict the binding of transcription factors and RNA-binding proteins and the structure of *CRHR1* mRNA. These tools use DNA/RNA sequences in FASTA format as input, and they output the results in the form of a sequence with highlighted protein-binding sites.

### Transcription factor binding

To predict allele-related alteration in transcription factor binding sites, we used the online tools PROMO (http://alggen.lsi.upc.es/cgibin/promo_v3/promo/promoinit.cgi?dirDB=TF_8.3) [34] and Ciiider (http://ciiider.com/) [35] with default parameters. The former tool (PROMO) uses positional weight matrices to construct transcription factor binding sites from taxonomically related species. The latter tool (Ciiider) predicts transcription factor binding sites from enrichment analysis of known regulatory regions.

### mRNA regulation

To predict allele-related alteration in RNA-binding protein sites, we used the RBPmap server (http://rbpmap.technion.ac.il/) with default parameters [36]. This tool maps RNA-binding proteins to predictably conserved RNA-binding sites. We also used the RNAfold program [37] to evaluate the potential effect of the 3′UTR rs28364021 variant on changing the *CRHR1* mRNA secondary structure (CACTGACAGCCTGGGGGGGCCGCTCT[C]CCCCTGCAGCCGTGCAGGACTCTAG-3′) with default parameters. We then analyzed the 3′UTR variant using the RNAComposer (http://rnacomposer.cs.put.poznan.pl/) [38] to predict the same sequence 3D structure and visualized the results with UCSF Chimera software (https://www.cgl.ucsf.edu/chimera/) by using default parameters [39].

## Results

Overall, we detected 122 unique *CRHR1* SNPs significantly linked to MDD and/or T2D: a total of 97 SNPs conferring only MDD risk, 22 SNPs conferring only T2D risk, and 3 SNPs conferring comorbid MDD-T2D risk. These SNPs were significantly linked/in LD (i.e., association) across different models (D1, D2, R1, and R2).

**Fig. 1:**
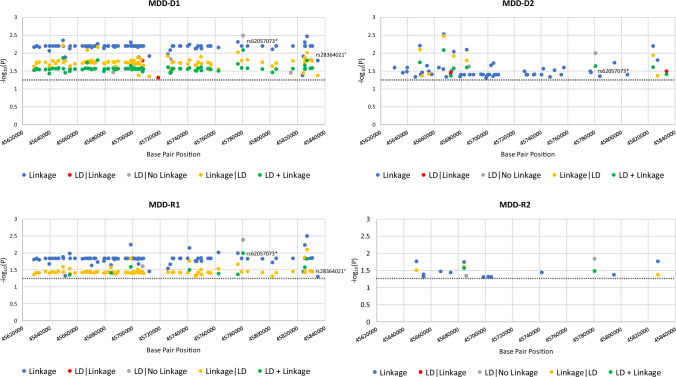
For each *CRHR1* risk SNPs and putative risk alleles in MDD, we present the -log10(P) as a function of each test statistic (Linkage, LD|Linkage, LD|NoLinkage, Linkage|LD, and LD + Linkage) and per inheritance model: D1: dominant, complete penetrance, D2: dominant, incomplete penetrance, R1: recessive, complete penetrance, R2: recessive, incomplete penetrance. (*variant predicted to affect transcription factor binding, °variant predicted to affect RNA-binding proteins and 3D RNA structure. The dotted line indicates statistical significance level

**Fig. 2:**
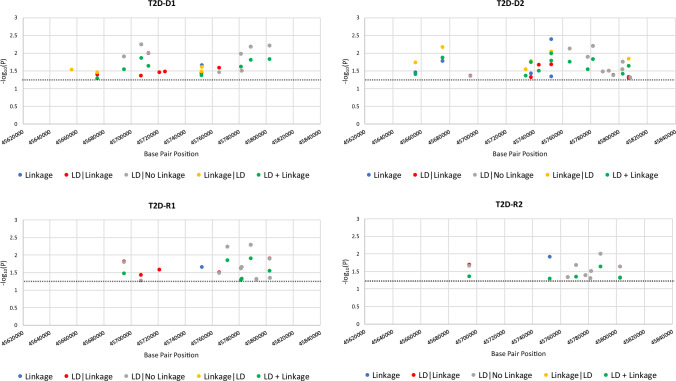
For each *CRHR1* risk SNPs and putative risk alleles in T2D, we present the -log10(P) as a function of each test statistic (Linkage, LD|Linkage, LD|NoLinkage, Linkage|LD, and LD + Linkage) and per inheritance model: D1: dominant, complete penetrance, D2: dominant, incomplete penetrance, R1: recessive, complete penetrance, R2: recessive, incomplete penetrance. The dotted line indicates statistical significance level

Specifically, we detected linkage to and/or LD (i.e., association): with MDD for 93 SNPs under the model D1, 57 SNPs under the model D2, 91 SNPs under the model R1, and 14 SNPs under the model R2 (Fig. 1); and with T2D for 15 SNPs under the model D1, 18 SNPs under the model D2, 12 SNPs under the model R1, and 10 SNPs under the model R2 (Fig. 2). Risk variants for MDD and T2D are shown among overlapping models (Supplementary Fig. 1A and 1B). While the MDD and T2D risk variants include major and minor alleles, the latter appear to prevail. Of the 97 MDD risk variants, 94 are novel, and of the 22 T2D risk variants, 19 are novel. Both novel and previously reported variants [40–43] are specified in Supplementary Tables I and II. The amplified and tested variants that were not significant either in MDD or in T2D are reported in Supplementary Table III.

**Fig. 3:**
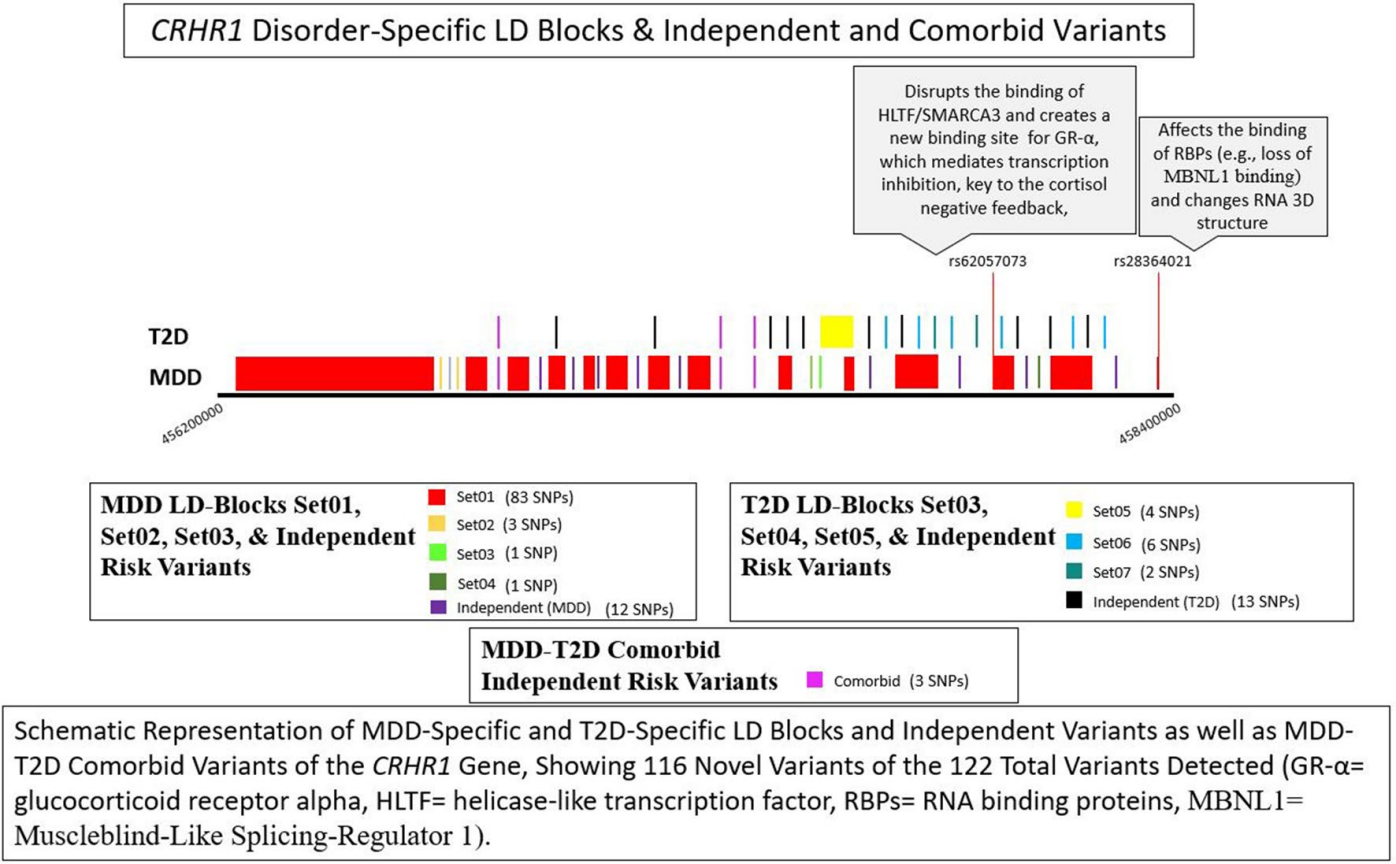
Schematic representation of MDD-specific and T2D-specific LD blocks and independent variants as well as MDD-T2D comorbid variants of the *CRHR1* gene, showing 116 novel variants of the 122 total variants detected (GR-α = glucocorticoid receptor alpha, HLTF = helicase-like transcription factor, RBPs = RNA-binding proteins, MBNL1 = muscleblind-like splicing regulator 1)

We report significant risk variants falling within LD blocks and identified 4 specific LD blocks (Set01, Set02, Set03, Set04) containing significant MDD risk variants (Supplementary Table I) and 3 additional specific LD blocks (Set05, Set06, Set07) containing significant T2D risk variants (Supplementary Table II). Set01, Set02, Set03, and Set04 contain 83, 3, 1, and 1 MDD risk variants, respectively, while Set05, Set06, and Set07 contain 4, 6, and 2 T2D risk variants, respectively (Fig. 3).

The detected LD blocks containing variants in linkage to and/or LD/association with MDD share none of the detected LD blocks containing variants in linkage to and/or LD/association with T2D. Among the independent SNPs, 3 appear to confer risk for MDD-T2D comorbidity. The remaining MDD risk variants and T2D risk variants are independent (Fig. 3). The variants in each specific LD block and the independent variants are shown in Supplementary Tables I and II.

## Results of in silico functional predictions

**Fig. 4:**
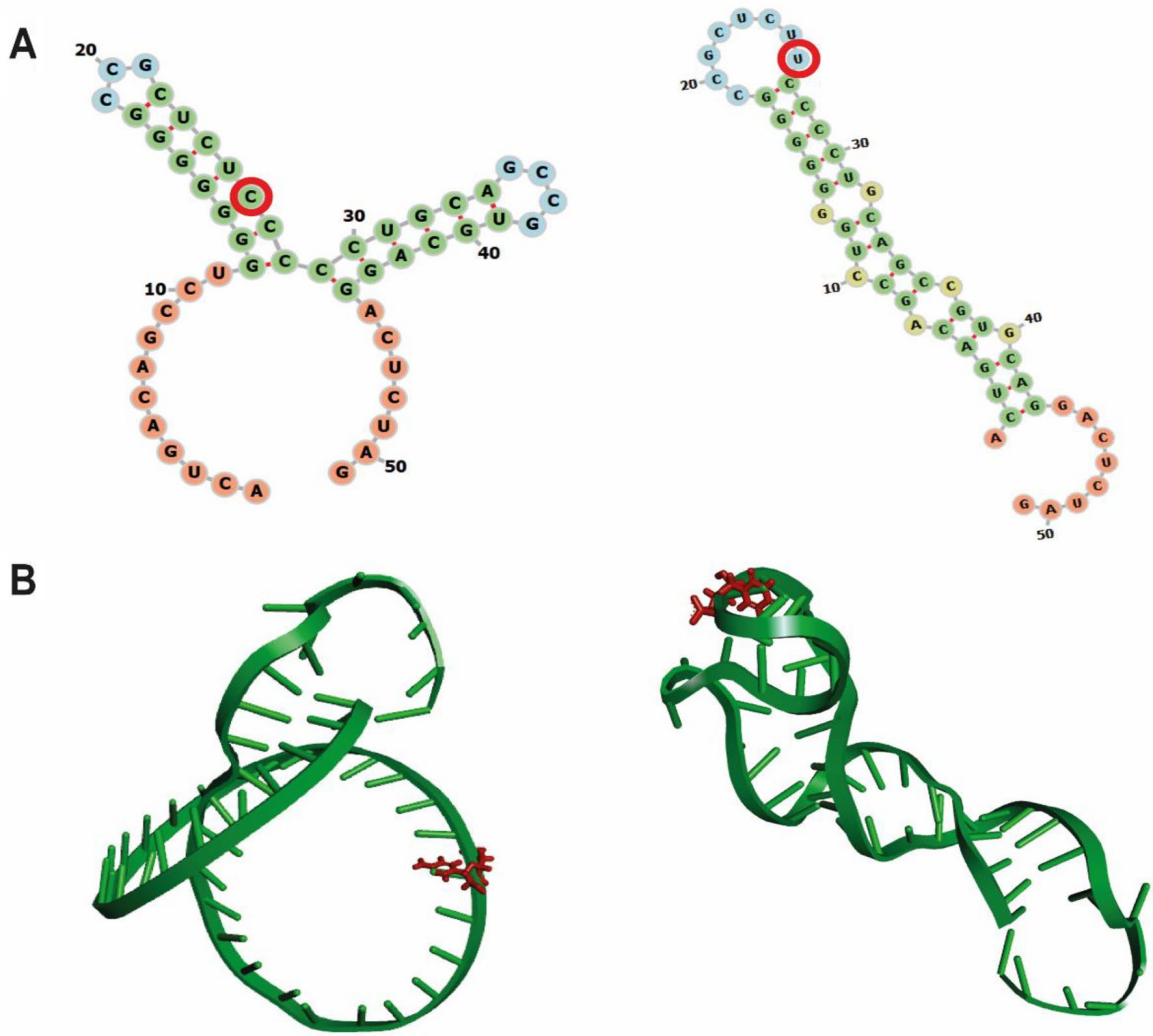
Structure models for the *CRHR1* rs28364021. A) RNA secondary structure models generated by Chimera (https://www.cgl.ucsf.edu/chimera) of the *CRHR1* 3′-UTR C (left) and T (right) alleles; the respective cytosine and thymine are circled in red for each structure. B) 3D model generated by RNAComposer of the *CRHR1* 3′-UTR C (left) and T (right) alleles; cytosine and thymine are in red for each structure

We found that the novel *CRHR1* rs62057073 MDD risk C-allele is predicted to disrupt the binding of the helicase-like transcription factor (HLTF, also known as SMARCA3) and creates a new binding site for the glucocorticoid receptor-alpha (GR-α) (Table 1). The *CRHR1* rs62057073 MDD risk C-allele belongs to the MDD-specific LD block Set01 largely represented within our MDD risk variants’ dataset. We also found that the novel variant *CRHR1* rs28364021 MDD-protective T-allele affects the binding site for poly-(RC)-binding protein 2 (PCBP2), polypyrimidine tract-binding protein 1 (PTBP1), serine- and arginine-rich splicing factor 2 (SRSF2), and serine- and arginine-rich splicing factor 3 (SRSF3) and creates a new binding site for muscleblind-like splicing regulator 1 (MBNL1), PTBP1, and SRSF3 proteins (Supplementary Table IV). The protective T-allele of the same risk variant was also predicted to decrease the minimum-free energy of *CRHR1* mRNA (CACTGACAGCCTGGGGGGGCCGCTCT[T]CCCCTGCAGCCGTGCAGGACTCTAG-3′) by  – 2.06 kcal/mol and the MDD risk C-nucleotide (reference allele) would form a canonical base pair within a stable stem loop (Fig. 4A); meanwhile, in contrast, the T-allele would not adopt a base pair in the structure. 3D modeling showed a difference in the structures when the either C-allele or T-allele is present. As in the secondary structure, the T-allele promotes a more stable structure (Fig. 4B). Of note, *CRHR1* rs28364021 MDD risk C-allele is included within the Set01 MDD-specific LD block largely identified in our MDD risk variants’ dataset.

**Table 1:** *CRHR1* rs62057073 predicted changes in transcription factor binding sites based on in silico studies

Nucleotide	Transcription factor	Binding site sequence
C	GR-α	C**C**TAG
	ENKTF-1	C**C**TAGCCA
	NFIX	**C**TAGCCAGC
T	ENKTF-1	CT**T**AGCCA
	NFIX	T**T**AGCCAGC
	HLTF/SMARCA3	CCTCT**T**AGCC

## Discussion

T2D with 6.28% prevalence worldwide [53] and MDD with 7.8% adult prevalence worldwide [54] are heterogeneous polygenic and complex disorders, and they are often comorbid [1, 2]. The serotonergic and HPA systems are both implicated in MDD [55] and may play a role in T2D [7, 56, 57]. As not all MDD patients develop T2D, and not all T2D patients have MDD, a genetic stratification in disease predisposition correlated to phenotype heterogeneity is expected. We focused on the *CRHR1* gene that has been related to the symptoms of depression and potentially contributes to increased risk for T2D.

In this study, we reported identifying in *CRHR1* 122 unique variants, of which 116 are novel, that are significantly linked/in LD (understanding that LD is association) with MDD, T2D, or both, across different modes of inheritance pattern. These variants appear to be more strongly related to MDD, although the families were primarily ascertained for T2D. We identified 3 novel risk variants (rs1706719, rs117267254, and rs1617406) significantly linked and/or associated with the risk of MDD-T2D comorbidity albeit 2 of these variants (rs1706719 and rs1617406) have different putative risk alleles in MDD and T2D, suggesting that the variants’ position and/or other epistatic interactions with the variants could mediate the comorbid risks. We identified a total of 7 LD blocks, 4 of which carry uniquely MDD risk variants (Set01, Set02, Set03, and Set04) and 3 of which carry uniquely T2D risk variants (Set05, Set06, and Set07). Risk variants reported within each block reciprocally confirmed the data validity within the block. The LD block Set01 was largely represented within the linkage variants with MDD rather than T2D. The LD block Set01 contained, among 83 MDD risk variants, 2 MDD risk variants (rs62057073 and rs28364021) with predicted functional significance. The novel rs62057073 variant corroborated the MDD phenotype through GR-α role implication and binding disruption of HLTF/SMARCA3. Of note, the GABAergic interneurons involved in depression express SMARCA3 and mediate the response to antidepressants through the p11/annexin A2/SMARCA3 complex. With chronic selective serotonin receptor inhibitor (SSRI) treatment, the p11/annexin A2/SMARCA3 complex mediates increased hippocampal neurogenesis and antidepressant responses; p11 knockout mice [44] and SMARCA3 knockout mice [45] show reduced behavioral and neurogenic responsiveness to SSRI therapy [46, 47]. These results should be analyzed in vitro and/or in vivo, possibly taking into consideration the whole LD block Set01 risk variants, whose epistatic interactions might dictate the final biological effects on *CRHR1*. As GR-α mediates the HPA axis negative feedback, and chronic stress may cause GR-α resistance and HPA axis negative feedback loss [48], the large intronic *CRHR1* risk region we detected as strongly implicated in MDD might impair *CRHR1* and represent a novel regulatory missing factor contributing to predisposition to depression and resistance to antidepressant therapy in some patients [49]. The novel rs28364021 variant corroborated the MDD phenotype through the predicted RNA-binding protein MBLN1 loss. Loss of MBNL proteins resulted in structural and morphological changes in brains of mice [50, 51] and significant differences in *MBNL1* gene expression were found in mice expressing anxiety–depression-like behavior [52]. Thus, PCBP2 and SRSF2 binding to *CRHR1* might confer risk for MDD, while MBNL1 binding to *CRHR1* might protect from MDD.

These risk variants probably drive the linkage in this region. However, experimental studies are needed to confirm these results. Two of the MDD risk variants in our study predisposed to adult depression in abused children, namely, rs173365 [41] and rs17689882 [42]; our MDD risk variant rs242941 predicted antidepressant response in Mexican–American children [40]. Interestingly, 3 T2D risk variants in our study were also previously reported in predisposition to depression in African-American children (rs7209436, rs242924) and Spanish children (rs110402) [41, 43]. This link supports our hypothesis of *CRHR1*-conferred genetic risk to T2D and MDD. However, these 3 variants appear in our dataset in a T2D-specific LD block (Set06); this might imply that ethnic differences may underlie the relationship between different LD blocks and disease and comorbidity.

**Fig. 5:**
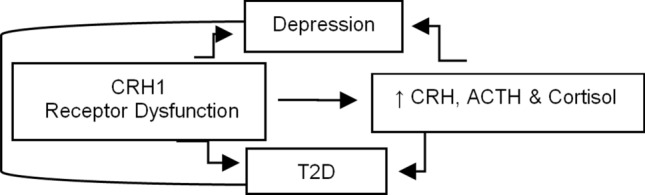
Hypothesized relationship between CRHR1 dysfunction, increased cortisol levels, depression, T2D

As the CRH system plays a role in depression-stress response [58, 59] and CRH SNPs are depression risk variants [59], and as *CRHR1* variants modulate antidepressant response [60–62], we hypothesize that CRH system resistance may lead to hypercortisolism (Fig. 5). In fact, hypercortisolemic patients with depression, if stimulated with CRH, show a blunted ACTH response [63], indicating that, in these patient subgroups, the cortisol-mediated negative feedback on the corticotroph cells is possibly normal. On the contrary, CRH infusion to healthy subjects reproduces the hypercortisolism of depression [64]. This difference suggests that hypercortisolism in depression may represent a defect at the CRHR level, at least in some patients, resulting in CRH hypersecretion and CRH-NE hyperactivation.

The most plausible explanation for MDD-T2D CRHR1-mediated comorbidity lies in the central limbic noradrenergic response to CRH, which triggers central NE [65], stimulating adrenal cortisol secretion [66, 67] and—by a positive feedback—hypothalamic and limbic CRH secretion, driving and maintaining the central HPA hyperactivation [68, 69]. While control subjects show plasma cortisol and cerebrospinal fluid (CSF) CRH levels being significantly negatively correlated, depressed patients have significantly higher circadian CSF NE and plasma cortisol levels; given their plasmatic hypercortisolism, they also have inappropriately “normal” plasma ACTH and CSF CRH [70]. These data strongly suggest that CRHR dysfunction is a possible basis for depression and hypercortisolism downstream effect may confer increased risk for T2D, thus supporting our hypothesis that *CRHR1* variants might potentially mediate, at least in part, the MDD-T2D comorbidity [70].

Further, hypercortisolism and altered feedback inhibition are HPA abnormalities of depression and T2D [71, 72], and T2D, metabolic traits, and MDD are associated with hypercortisolemia (Fig. 5) [7, 12]. As stress responses lead to hypercortisolism, they might also lead to T2D. Aging, per se, is associated with both the HPA axis’ reduced feedback and hyperactivity [15], which may lead to T2D, metabolic traits, and depression. High cortisol increases glycogenolysis, gluconeogenesis, and insulin resistance in the liver and muscle both indirectly (via increased lipolysis and proteolysis, thus increased fatty acids and amino acids, respectively) and directly (via counteraction on the insulin receptor) and increases visceral obesity and decreases insulin secretion; all of these effects, per se, can jointly lead to T2D and metabolic traits [73]. Several HPA axis receptor genes are associated with metabolic abnormalities [74, 75]. The known physiologic effects of glucocorticoids suggest that inherited predisposition to HPA axis activation and high cortisol may contribute to glucose intolerance [73], metabolic traits, and depressive symptoms [7]. Increased cortisol causes serotonergic dysfunction, a substrate for depression [8]. A study in T2D patients reported that the prevalence of subclinical hypercortisolism was significantly higher in T2D patients than in control subjects, with a 7% proportion of T2D statistically attributable to subclinical hypercortisolism. Furthermore, subclinical hypercortisolism was significantly associated with severe T2D, defined by the presence of insulin treatment, hypertension, and dyslipidemia [76]. In African-American adults with and without T2D, long-term HPA axis dysregulation represented by high hair cortisol is associated with increased glycosylated hemoglobin (HbA1c) in the whole group and T2D group [17].

This study stemmed from the need to stratify the genetic risk conferred by the cortisol pathway and correlate it with T2D and MDD. The correlation of *CRHR1* gene with the studied phenotype(s) begins to elucidate the genetic basis of the T2D association with MDD and may provide the foundation upon which other data and pathogenic hypotheses will subsequently be built. The goal is to implement preventive plans targeting subjects identified as at risk for T2D and MDD, who may, by knowing their genetic make-up and risk for such disorders, be inclined to undergo behavioral-cognitive therapy and lifestyle modifying behaviors. Such preventive plans may significantly decrease the burden of T2D–MDD comorbidity on patients and on the healthcare system. In the long term, we may succeed in therapeutically targeting the CRHR1 implicated in the HPA axis disruption in subjects with family risk for T2D and MDD, thereby reversing the HPA axis hyperactivity to a physiological state and preventing both disorders.

We have challenged the currently accepted paradigm that T2D is only a metabolic disorder and that depression is purely a mental disorder; we showed that they are, at least partially and/or in a subgroup of patients, associated with *CRHR1* variants, likely conferring predisposition to hyperactivity of the neuro-endocrine glucocorticosteroid pathway. Our study is innovative as there are no extensive data regarding genetic screening of the *CRHR1* gene jointly investigating T2D and depression traits in humans. Performing linkage and LD tests not only in a group of patients with T2D, but also with MDD, is a powerful and innovative strategy for gene identification. This study stems from the hypothesis that T2D does not merely reflect a dysmetabolic disorder, affecting blood glucose and lipid levels and blood pressure. Rather it is a complex disorder characterized by increased predisposition to stress-related hyperactivity and potentially abnormal psychological traits leading to abnormal behavioral and compensatory mechanisms such as stress-induced eating [77].

We have explored a new avenue in the human genetics of T2D with MDD: the impact of single gene variants and LD blocks in two major, apparently distinct disorders (i.e., T2D and depression). Our investigation of the LD blocks within the 2 phenotypes proves studies are needed regarding the risk effects of gene variants in comorbid patients and families (Fig. 3).

There are several limitations to this study. First the employed genotyping methodology (microarray) is only indicative of tested variants and actual nearby risk variants could either be inferred by the presence of additional variants in LD or known by direct sequencing. Second, the population in our study is mono-ethnic and further studies are needed to replicate our findings in other ethnic groups.

In summary, we used an interdisciplinary approach of human genetics and clinical phenotyping of T2D with MDD and pioneered the joint study of a neuro-endocrine stress-related gene, *CRHR1*, in human T2D and MDD, a metabolic and mental disorder, whose pathogenesis might be shared. These results will prompt new research in the area of associated mental and metabolic disorders, thereby creating a new focus on the neuro-endocrine-mental-metabolic dysfunctions that may characterize pre-disease states. Opening our eyes to the idea of this joint pathogenesis of such apparently different disorders will dramatically advance the research in the field of both diseases, as well as bring major benefits to the understanding and management of both disorders.

## Availability of data

The data presented in this study are available on reasonable request. The data are not publicly available due to privacy restrictions, lacking specific patients’ consent.

## Supplementary Information

Below is the link to the electronic supplementary material.

Supplementary file1 (DOCX 51 KB) Supplementary Table I: *CRHR1*-risk SNPs for MDD; and Supplementary Table II: *CRHR1*-risk SNPs for T2D.

Supplementary file2 (XLSX 9 KB) Supplementary Table III: All *CRHR1* variants amplified and tested that were not significant either in MDD or in T2D.

Supplementary file3 (DOCX 15 KB) Supplementary Table IV: In Silico RNA-binding protein sites generated by *CRHR1* 3’-UTR rs28364021 C>T alleles.

Supplementary file4 (PDF 1341 KB) Supplementary Figure 1A: A Venn diagram with the number of the overlapping SNPs among the 4 parametric models in MDD; and Supplementary Figure 1B: A Venn diagram with the number of the overlapping SNPs among the 4 parametric models in T2D.

## Abbreviations

ACTH: Adrenocorticotropin hormone
CRH: Corticotrophin-releasing hormone (alias corticotropin-releasing factor, CRF)
CRH-NE-CRH: CRH-norepinephrine (NE)-CRH system
CRHR: Corticotropin-releasing hormone receptor
CRHR1: Corticotropin-releasing hormone receptor 1
CRHR2: Corticotropin-releasing hormone receptor 2
CSF: Cerebrospinal fluid
GR-α: Glucocorticoid receptor-alpha
HbA1c: Glycosylated hemoglobin
HLTF: Helicase-like transcription factor (also known as SMARCA3)
HPA axis: Hypothalamic–pituitary–adrenal axis
LD: Linkage disequilibrium
MBNL1: Muscleblind-like splicing regulator 1
MDD: Major depressive disorder
PCBP2: Poly-(RC)-binding protein 2
PTBP1: Polypyrimidine tract-binding protein 1
RBPs: RNA-binding proteins sites
SNPs: Single-nucleotide polymorphisms
SRSF2: Serine- and arginine-rich splicing factor 2
SRSF3: Serine- and arginine-rich splicing factor 3
SSRI: Selective serotonin receptor inhibitor
T2D: Type 2 diabetes
