# Supplementary material for: LD block disorder-specific pleiotropic roles of novel CRHR1 in type 2 diabetes and depression disorder comorbidity
Source: Eur Arch Psychiatry Clin Neurosci. 2023 Dec 14;275(4):1025–35. doi: 10.1007/s00406-023-01710-x (PMC12148968; doi:10.1007/s00406-023-01710-x)
Supplement: Supplementary file 1 — Supplementary file1 (DOCX 51 KB) Supplementary Table I: CRHR1-risk SNPs for MDD; and Supplementary Table II: CRHR1-risk SNPs for T2D. [file 406_2023_1710_MOESM1_ESM.docx]

**Supplementary Table I**. *CRHR1*-risk SNPs for MDD

Supplementary Table I All SNPs significant in MDD, the parametric model under which the significance was detected, the chromosomal 17 base pair sequence position, the Ref and Alt alleles, the minimum allele frequency (MAF) in our dataset, the risk allele, the location or consequence of the variants, the LD block detected or their independent status, and whether they were novel or previously published in MDD or T2D. Comorbid SNPs are highlighted in bold.

| **Model^1^** | **SNP** | **Position** | **Ref** | **Alt** | **MAF** | **Risk Allele** | **Consequence** | **LD Block** | **Reference** |
| --- | --- | --- | --- | --- | --- | --- | --- | --- | --- |
| D1,R1 | rs365825 | 45628235 | A | G | 0.126 | A | Intronic | Set01 |  |
| D1,R1 | rs681485 | 45630253 | C | A | 0.124 | C | Intronic | Set01 |  |
| D1,D2,R1 | rs796835241 | 45630254 | G | A | 0.122 | G | Intronic | Set01 |  |
| D1,R1 | rs436667 | 45632049 | C | T | 0.122 | C | Intronic | Set01 |  |
| D1 | rs241041 | 45636559 | A | C | 0.124 | A | Intronic | Set01 |  |
| D1 | rs413778 | 45639519 | A | G | 0.129 | A | Splice-site | Set01 |  |
| D1,D2,R1 | rs389217 | 45639765 | C | A | 0.122 | C | Intronic | Set01 |  |
| D1 | rs393152 | 45641777 | A | G | 0.123 | A | Intronic | Set01 |  |
| D1,R1 | rs2942164 | 45643917 | G | C | 0.123 | G | Intronic | Set01 |  |
| D1,D2,R1 | rs413917 | 45645823 | G | A | 0.123 | G | Intronic | Set01 |  |
| D1 | rs3418 | 45646096 | C | A | 0.124 | C | Intronic | Set01 |  |
| D1,D2,R1,R2 | rs439945 | 45649293 | A | C | 0.148 | A | Intronic | Set02 |  |
| D1,D2,R1 | rs453997 | 45649695 | C | A | 0.123 | C | Intronic | Set01 |  |
| D1,D2,R1 | rs417968 | 45651010 | G | A | 0.150 | G | Intronic | Set02 |  |
| D1,R1,R2 | rs241036 | 45654353 | A | C | 0.124 | A | Intronic | Set01 |  |
| D1,D2,R1,R2 | rs241035 | 45654530 | C | A | 0.123 | C | Intronic | Set01 |  |
| D2 | **rs1706719** | 45655898 | C | T | 0.026 | C | Intronic | Independent |  |
| D1,D2,R1 | rs241027 | 45658112 | A | C | 0.124 | A | Intronic | Set01 |  |
| D1,D2,R1 | rs62053939 | 45663490 | C | T | 0.121 | C | Intronic | Set01 |  |
| D1,R1 | rs17760631 | 45665679 | T | C | 0.123 | T | Intronic | Set01 |  |
| D2 | rs62053943 | 45666837 | C | A | 0.080 | C | Intronic | Independent |  |
| D1,D2,R1,R2 | rs1635298 | 45666978 | T | A | 0.137 | T | Intronic | Set01 |  |
| D1,R1 | rs17687462 | 45667624 | C | A | 0.123 | C | Intronic | Set01 |  |
| D1,D2,R1 | rs17760733 | 45668910 | G | T | 0.122 | G | Intronic | Set01 |  |
| D1,R1 | rs17687504 | 45669355 | A | C | 0.123 | A | Intronic | Set01 |  |
| D1,R1 | rs1724407 | 45670249 | C | A | 0.146 | C | Intronic | Set01 |  |
| D1,R1 | rs62053953 | 45672088 | G | A | 0.123 | G | Intronic | Set01 |  |
| D2 | rs118190895 | 45672328 | T | C | 0.002 | C | Intronic | Independent |  |
| D1 | rs17687625 | 45672872 | A | C | 0.124 | A | Intronic | Set01 |  |
| D1,D2,R1,R2 | rs1635291 | 45674547 | G | A | 0.141 | G | Intronic | Set01 |  |
| D1,D2,R1 | rs757502 | 45679140 | C | A | 0.117 | C | Intronic | Set01 |  |
| D1,R1 | rs757501 | 45679319 | A | C | 0.122 | A | Intronic | Set01 |  |
| D1,D2,R1 | rs78487840 | 45679795 | C | T | 0.123 | C | Intronic | Set01 |  |
| D1,R1 | rs76453925 | 45682065 | C | A | 0.123 | C | Intronic | Set01 |  |
| D1,D2,R1 | rs17687849 | 45682353 | A | C | 0.123 | A | Intronic | Set01 |  |
| D2,R1,R2 | rs34240571 | 45684385 | G | A | 0.007 | G | Intronic | Independent |  |
| D1,R1 | rs17688032 | 45685836 | G | A | 0.123 | G | Intronic | Set01 |  |
| D1,D2,R1,R2 | rs117849721 | 45685926 | G | T | 0.005 | G | Intronic | Independent |  |
| D1,D2,R1 | rs17688068 | 45686569 | A | C | 0.122 | A | Intronic | Set01 |  |
| D1,R1 | rs77220413 | 45687012 | G | A | 0.123 | G | Intronic | Set01 |  |
| D1,R1 | rs17688126 | 45687714 | A | C | 0.124 | A | Intronic | Set01 |  |
| D1,D2,R1 | rs17688249 | 45689388 | C | A | 0.122 | C | Intronic | Set01 |  |
| D1,D2,R1 | rs17688410 | 45694885 | C | A | 0.123 | C | Intronic | Set01 |  |
| D1,D2,R1 | rs17688558 | 45696577 | A | C | 0.122 | A | Intronic | Set01 |  |
| D1,D2,R1,R2 | rs12150454 | 45698695 | C | A | 0.124 | C | Intronic | Set01 |  |
| D1,R1 | rs12150091 | 45698876 | A | C | 0.123 | A | Intronic | Set01 |  |
| D1,D2,R1 | rs17761985 | 45699421 | C | A | 0.122 | C | Intronic | Set01 |  |
| D1,D2,R1 | rs17688767 | 45700176 | A | C | 0.122 | A | Intronic | Set01 |  |
| D1,R1 | rs17688875 | 45701040 | A | C | 0.123 | A | Intronic | Set01 |  |
| D1,D2,R1 | rs17762165 | 45701236 | C | A | 0.122 | C | Intronic | Set01 |  |
| D1,D2,R1,R2 | rs1526128 | 45702258 | C | A | 0.120 | C | Intronic | Set01 |  |
| D1,R1 | rs968028 | 45703739 | A | C | 0.128 | A | Intronic | Set01 |  |
| D1,D2,R1 | rs968027 | 45703884 | C | A | 0.123 | C | Intronic | Set01 |  |
| D1,D2,R1,R2 | rs17762361 | 45704412 | A | C | 0.122 | A | Intronic | Set01 |  |
| D1,D2,R1 | rs17689104 | 45705126 | A | C | 0.124 | A | Intronic | Set01 |  |
| D1,D2,R1 | rs17689182 | 45706207 | C | A | 0.122 | C | Intronic | Set01 |  |
| R1 | **rs117267254** | 45707259 | G | A | 0.029 | G | Intronic | Independent |  |
| D1,R1 | **rs1617406** | 45707411 | G | A | 0.107 | A | Intronic | Independent |  |
| D1,D2,R1 | rs61667602 | 45707983 | T | C | 0.123 | T | Intronic | Set01 |  |
| D1,R1 | rs62056931 | 45712332 | G | A | 0.120 | G | Intronic | Set01 |  |
| D1 | rs2864088 | 45718813 | T | C | 0.014 | C | Intronic | Set03 |  |
| D1,R1 | rs1358071 | 45725823 | C | A | 0.141 | C | Intronic | Set02 |  |
| D1,D2,R1 | rs56380663 | 45727253 | C | T | 0.123 | C | Intronic | Set01 |  |
| D1 | rs9303521 | 45727828 | T | G | 0.241 | G | Intronic | Independent |  |
| D1,D2,R1 | rs1880752 | 45728898 | C | A | 0.123 | C | Intronic | Set01 |  |
| D1,D2,R1 | rs2864087 | 45729697 | C | A | 0.122 | C | Intronic | Set01 |  |
| D1 | rs17563718 | 45734307 | C | A | 0.122 | C | Intronic | Set01 |  |
| D1,D2,R1 | rs1526126 | 45734852 | C | A | 0.123 | C | Intronic | Set01 |  |
| D1,D2,R1 | rs17563800 | 45740093 | C | T | 0.121 | C | Intronic | Set01 |  |
| D1,D2,R1,R2 | rs17563861 | 45741540 | A | C | 0.124 | A | Intronic | Set01 |  |
| D1,R1 | rs76171147 | 45745863 | T | C | 0.125 | T | Intronic | Set01 |  |
| D1,D2,R1 | rs62054431 | 45746514 | C | A | 0.123 | C | Intronic | Set01 |  |
| D1,R1 | rs12150332 | 45747542 | C | A | 0.122 | C | Intronic | Set01 |  |
| D1,D2,R1 | rs17334923 | 45749878 | C | A | 0.125 | A | Intronic | Set01 |  |
| D1,R1 | rs12150455 | 45750142 | A | C | 0.125 | A | Intronic | Set01 |  |
| D1,D2,R1 | rs35631660 | 45754971 | A | C | 0.122 | A | Intronic | Set01 |  |
| D1,R1 | rs34579278 | 45756615 | A | C | 0.123 | A | Intronic | Set01 |  |
| D1,D2,R1 | rs34008514 | 45756948 | A | C | 0.123 | A | Intronic | Set01 |  |
| D1,R1 | rs11079719 | 45762640 | T | A | 0.119 | T | Intronic | Set01 |  |
| D1,D2,R1 | rs56303031 | 45776556 | G | A | 0.124 | A | Intronic | Set01 |  |
| D2, | rs34186148 | 45777289 | G | C | 0.205 | G | Intronic | Independent |  |
| D1,R1 | rs62057061 | 45779273 | C | G | 0.124 | C | Intronic | Set01 |  |
| D1,D2,R1,R2 | rs117910967 | 45780445 | C | A | 0.005 | A | Intronic | Independent |  |
| D1,D2,R1 | rs62057073 | 45783751 | C | T | 0.120 | C | Intronic | Set01 |  |
| D1,D2,R1,R2 | rs56319902 | 45794616 | C | T | 0.121 | T | Intronic | Set01 |  |
| D1,R1 | rs80184151 | 45801942 | A | G | 0.126 | A | Intronic | Set01 |  |
| D1,D2,R1 | rs17689378 | 45804424 | C | T | 0.123 | C | Intronic | Set01 |  |
| D1 | rs242941 | 45815154 | A | C | 0.173 | A | Intronic | Independent | (73) |
| D1,R1 | rs28364023 | 45816793 | C | A | 0.124 | C | Intronic | Set01 |  |
| D1,D2 | rs173365 | 45823708 | A | C | 0.222 | C | Intronic | Set04 | (71) |
| D1,R1 | rs62057144 | 45824192 | A | G | 0.124 | A | Intronic | Set01 |  |
| D1,R1 | rs3885075 | 45825433 | A | C | 0.124 | A | Intronic | Set01 |  |
| D1,R1 | rs41280116 | 45825476 | C | A | 0.124 | C | Intronic | Set01 |  |
| D1,R1 | rs1912151 | 45825578 | C | A | 0.124 | C | Intronic | Set01 |  |
| D1,R1 | rs41280118 | 45825723 | C | A | 0.123 | C | Intronic | Set01 |  |
| D1,D2,R1,R2 | rs17689824 | 45827031 | C | A | 0.124 | C | Intronic | Set01 |  |
| D1,R1 | rs17689882 | 45829462 | G | A | 0.123 | G | Intronic | Set01 | (72) |
| D1,R1 | rs16940671 | 45830785 | C | A | 0.125 | C | Intronic | Set01 |  |
| D2 | rs199643306 | 45833504 | G | A | 0.002 | G | Missense | Independent |  |
| D1,R1 | rs28364021 | 45834916 | C | T | 0.121 | C | UTR-3 | Set01 |  |

^1^Models: D1: Dominant complete-penetrance, D2: dominant incomplete-penetrance, R1: Recessive complete-penetrance, R2: Recessive incomplete-penetrance

**Supplementary Table II**. *CRHR1*-risk SNPs for T2D

Supplementary Table II All SNPs significant in T2D, the parametric model under which the significance was detected, the chromosomal 17 base pair sequence position, the Ref and Alt alleles, the minimum allele frequency (MAF) in our dataset, the risk allele, the location or consequence of the variants, the LD block detected or their independent status, and whether they were novel or previously published in MDD or T2D. Comorbid SNPs are highlighted in bold.

| **Model^1^** | **SNP** | **Position** | **Ref** | **Alt** | **MAF** | **Risk Allele** | **Consequence** | **LD Block** | **Reference** |
| --- | --- | --- | --- | --- | --- | --- | --- | --- | --- |
| D1,D2 | **rs1706719** | 45655898 | C | T | 0.026 | T | Intronic | Independent |  |
| D1,D2 | rs117536416 | 45674939 | C | G | 0.017 | C | Intronic | Independent |  |
| D1,D2,R1,R2 | rs16941058 | 45694724 | A | G | 0.043 | A | Intronic | Independent |  |
| D1,R1 | **rs117267254** | 45707259 | G | A | 0.029 | G | Intronic | Independent |  |
| D1 | **rs1617406** | 45707411 | G | A | 0.107 | G | Intronic | Independent |  |
| D1 | rs11871395 | 45712690 | G | A | 0.114 | A | Intronic | Independent |  |
| D1,R1 | rs117615688 | 45720942 | G | A | 0.044 | G | Intronic | Independent |  |
| D1 | rs7218457 | 45725140 | A | C | 0.190 | C | Intronic | Independent |  |
| D2 | rs1880753 | 45733894 | G | A | 0.250 | A | Intronic | Set05 |  |
| D2 | rs7220839 | 45737779 | C | A | 0.249 | A | Intronic | Set05 |  |
| D2 | rs8075654 | 45743303 | G | A | 0.234 | A | Intronic | Set05 |  |
| D1,D2 | rs1880755 | 45752038 | C | A | 0.246 | A | Intronic | Set05 |  |
| D1,D2,R1,R2 | rs17335035 | 45752192 | A | G | 0.012 | G | Intronic | Independent |  |
| D1,D2,R1,R2 | rs6503448 | 45765107 | G | A | 0.294 | G | Intronic | Set06 |  |
| R1,R2 | rs7225082 | 45771129 | G | A | 0.191 | G | Intronic | Independent |  |
| D2,R2 | rs4074461 | 45777790 | G | A | 0.289 | G | Intronic | Set06 |  |
| D1,R1,R2 | rs12942254 | 45781339 | C | A | 0.080 | A | Intronic | Set07 |  |
| D1,D2,R1,R2 | rs12950522 | 45781740 | A | C | 0.288 | C | Intronic | Set06 |  |
| D1,D2,R1,R2 | rs12940065 | 45788505 | G | A | 0.085 | A | Intronic | Set07 |  |
| D2,R1 | rs7209436 | 45792776 | C | T | 0.320 | T | Intronic | Set06 | (71) |
| D2 | rs62057097 | 45795918 | C | T | 0.075 | C | Intronic | Independent |  |
| D1,D2,R1,R2 | rs16940646 | 45802545 | A | C | 0.049 | C | Intronic | Independent |  |
| D2,R1,R2 | rs110402 | 45802681 | G | A | 0.314 | A | Intronic | Set06 | (74) |
| D2 | rs12936511 | 45807036 | C | T | 0.024 | C | Synonymous | Independent |  |
| D2 | rs242924 | 45808001 | G | A | 0.311 | A | Intronic | Set06 | (71) |

^1^Models: D1: Dominant complete-penetrance, D2: dominant incomplete-penetrance, R1: Recessive complete-penetrance, R2: Recessive incomplete-penetrance
