# Supplementary material for: LD block disorder-specific pleiotropic roles of novel CRHR1 in type 2 diabetes and depression disorder comorbidity
Source: Eur Arch Psychiatry Clin Neurosci. 2023 Dec 14;275(4):1025–35. doi: 10.1007/s00406-023-01710-x (PMC12148968; doi:10.1007/s00406-023-01710-x)
Supplement: Supplementary file 3 — Supplementary file3 (DOCX 15 KB) Supplementary Table IV: In Silico RNA-binding protein sites generated by CRHR1 3’-UTR rs28364021 C>T alleles. [file 406_2023_1710_MOESM3_ESM.docx]

**Supplementary Table IV.** *In Silico* RNA-binding protein sites generated by *CRHR1* 3’-UTR rs28364021 C>T alleles

Supplementary Table IV The in silico RNA-binding protein sites generated by CRHR1 3’-UTR rs28364021 C>T alleles, whereas C represents the MDD risk allele and T the MDD-protective allele.

| **Protein** | **Motif** | **3’-UTR sequences** | **P-value** |
| --- | --- | --- | --- |
| **MDD-risk C-allele** |  |  |  |
| **PCBP2** | ccyycch | ggccgcucuCccccug | 2.87E-02 |
|  |  | ggccgcucuCccccug | 2.79E-02 |
| **PTBP1** | Cucucu | ggccgcucuCccccug | 4.00E-03 |
|  |  | ggccgcucuCccccug | 1.76E-02 |
|  |  | ggccgcucuCccccug | 1.76E-02 |
| **SRSF2** | ugcygyy | ggccgcucuCccccug | 1.74E-0.2 |
|  |  | ggccgcucuCccccug | 2.27E-02 |
| **SRSF3** | cuckucy | ggccgcucuCccccug | 5.08E-03 |
|  |  | ggccgcucuCccccug | 1.32E-02 |
| **MDD-protective T-allele** |  |  |  |
| **MBNL1*** | Ygcuky | ggccgcucuUccccug | 8.38E-03 |
| **PTBP1** | Ucuu | ggccgcucuUccccug | 1.53E-02 |
| **SRSF3** | cuckucy | ggccgcucuUccccug | 4.65E-02 |
|  | Wcwwc | ggccgcucuUccccug | 2.61E-02 |
|  | cuckucy | ggccgcucuUccccug | 3.14E-02 |

*Novel binding

Red letters are the sites identified by the RBPmap program; the capital letters correspond to the position of the variant. The sites are reported as predicted binding sites if their P-value < 0.05. y= C or T; h= A or C or T; K= G or T; w= A or T.
