# Supplementary material for: LD block disorder-specific pleiotropic roles of novel CRHR1 in type 2 diabetes and depression disorder comorbidity
Source: Eur Arch Psychiatry Clin Neurosci. 2023 Dec 14;275(4):1025–35. doi: 10.1007/s00406-023-01710-x (PMC12148968; doi:10.1007/s00406-023-01710-x)
Supplement: Supplementary file 4 — Supplementary file4 (PDF 1341 KB) Supplementary Figure 1A: A Venn diagram with the number of the overlapping SNPs among the 4 parametric models in MDD; and Supplementary Figure 1B: A Venn diagram with the number of the overlapping SNPs among the 4 parametric models in T2D. [file 406_2023_1710_MOESM4_ESM.pdf]

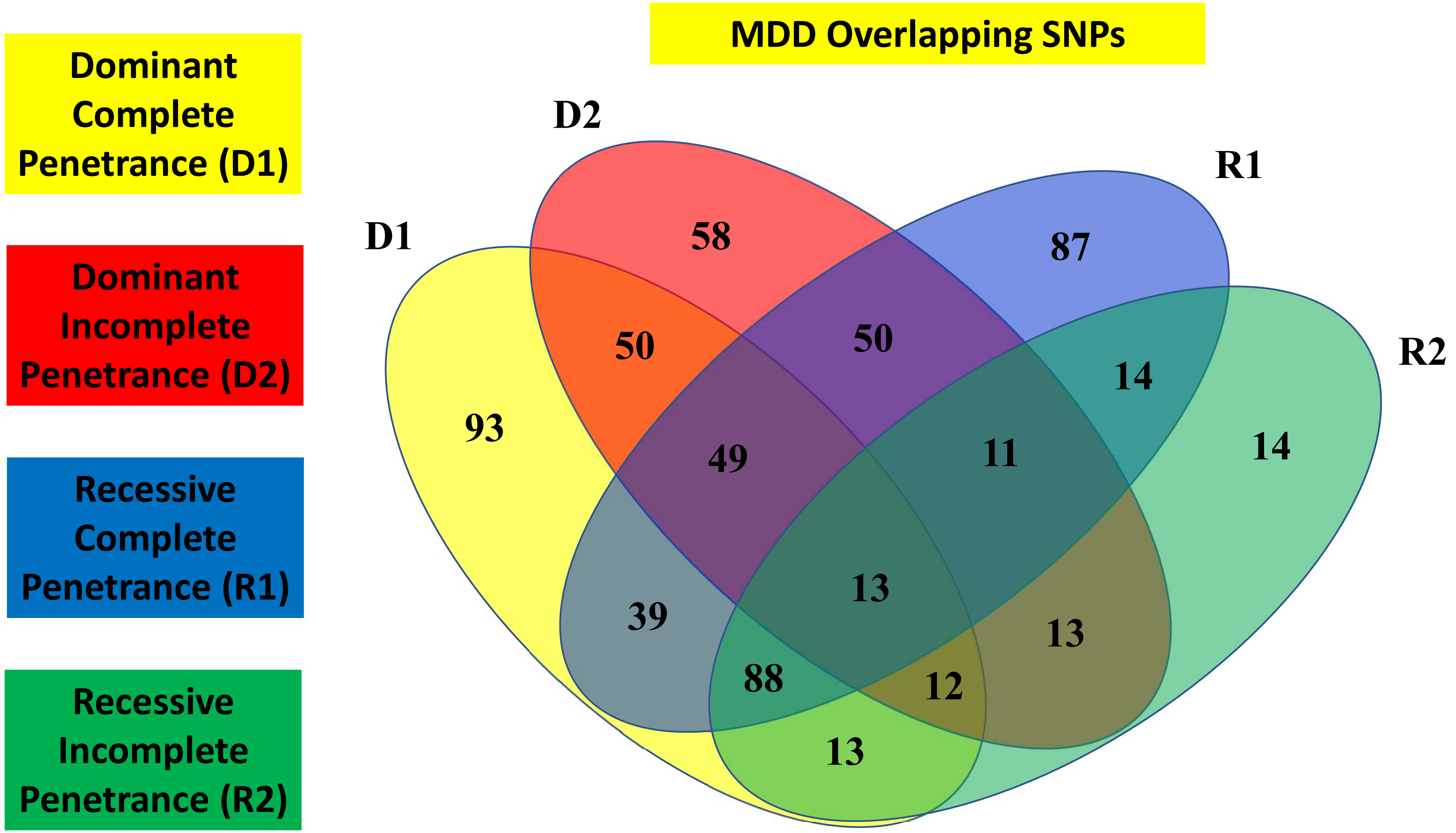

Supplemental Figure 1A: MDD. Overlapping models for *CRHR1*-Risk SNPs in MDD using a Venn diagram.

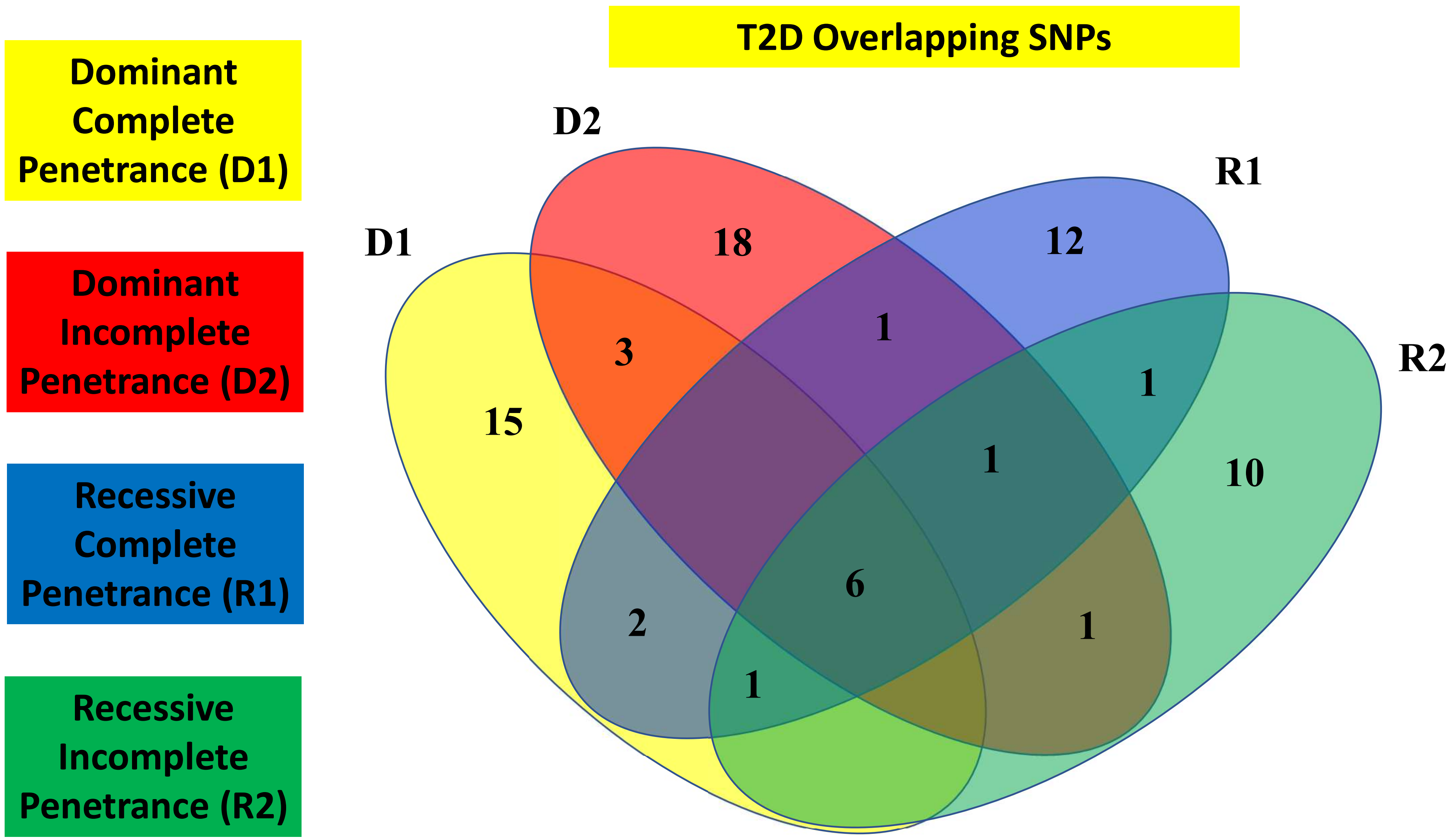

Supplemental Figure 1B: T2D. Overlapping models for *CRHR1*-Risk SNPs in T2D using a Venn diagram.
